# *MeNADP-ME3* Confers Salt and Drought Tolerance in *Arabidopsis* and Drives Functional Diversification of the *NADP-ME* Family in Cassava

**DOI:** 10.3390/cimb48030331

**Published:** 2026-03-20

**Authors:** Shuwen Wu, Zhanming Xia, Jiazheng Zhao, Changyi Wang, Yi Min, Dayong Wang

**Affiliations:** 1 Department of Biotechnology, School of Life and Health Sciences, Hainan University, Haikou 570228, China; 2 Laboratory of Biopharmaceuticals and Molecular Pharmacology, School of Pharmaceutical Sciences, Hainan University, Haikou 570228, China; 3 One Health Cooperative Innovation Center, Hainan University, Haikou 570228, China; 4 Key Laboratory of Tropical Biological Resources, Hainan University, Haikou 570228, China

**Keywords:** abiotic stress, C3-C4 intermediate species, cassava, *MeNADP-ME3*, NADP-ME, ScRNA-seq

## Abstract

As a typical C3-C4 intermediate plant, cassava (*Manihot esculenta* Crantz) exhibits high photosynthetic efficiency and low photorespiration. NADP-malic enzyme (NADP-ME) is a key enzyme in the C4 photosynthetic pathway that provides elevated ${\mathrm{CO}}_{2}$ concentrations for Rubisco. However, research on NADP-ME in C3-C4 intermediate species remains limited. In this study, we identified four *NADP-ME* genes in the cassava genome, with segmental duplication serving as the primary driving force for gene evolution. Cis-acting element analysis indicated potential roles of *MeNADP-ME* genes in environmental adaptation, stress responses, and growth regulation. Expression profiling using bulk RNA sequencing and single-cell RNA sequencing revealed distinct expression patterns in different tissues and cell subsets. Comparative analysis with *Arabidopsis* (*Arabidopsis thaliana*) and maize (*Zea mays*) NADP-ME families demonstrated that *MeNADP-ME3* exhibits bundle sheath cell-specific expression analogous to *ZmchlC4NADP-ME* in maize. Notably, photosynthetic genes and plasmodesmata (PD)-related genes exhibited high co-expression within mesophyll subcluster 13 and bundle sheath cells, providing molecular evidence for a limited C4 photosynthetic pathway in cassava. Protein–protein interaction predictions implicated MeNADP-ME3 in photosynthetic carbon metabolism and photorespiration regulation. Furthermore, qRT-PCR revealed significant responsiveness of *MeNADP-ME3* to various abiotic stresses, and confocal imaging confirmed its chloroplast localization. Functional validation demonstrated that *Arabidopsis* overexpressing *MeNADP-ME3* exhibited 30–120% enhanced antioxidant enzyme activities (SOD, POD, CAT) and 20–32% reduced oxidative damage markers (MDA, $H_{2}O_{2}$) under drought and salt stresses. These findings reveal the evolutionary trajectory of *NADP-ME* genes in C3-C4 intermediate species and provide genetic resources for developing stress-tolerant cassava cultivars.

## 1. Introduction

Cassava (*Manihot esculenta* Crantz) is recognized as a vital crop for addressing food security challenges and bioenergy development [1,2,3]. Compared to C3 and C4 plants, cassava features high photosynthetic efficiency and low photorespiration. Despite lacking Kranz anatomy, its phosphoenolpyruvate carboxylase (PEPC) activity exceeds that of C3 plants [4,5], supporting its classification as a C3-C4 intermediate species [6]. In C4 plants, PEPC initially fixes ${\mathrm{CO}}_{2}$ into C4 acids within mesophyll cells, which subsequently diffuse via plasmodesmata (PD) to bundle sheath cells for decarboxylation [7]. Subsequently, NADP-malic enzyme (NADP-ME; EC 1.1.1.40) catalyzes malate decarboxylation in bundle sheath cells, producing pyruvate, ${\mathrm{CO}}_{2}$, and NADPH, thereby increasing the ${\mathrm{CO}}_{2}$ concentration available for ribulose-1,5-bisphosphate carboxylase/oxygenase (Rubisco) [8]. This unique ${\mathrm{CO}}_{2}$ concentrating mechanism (CCM) in C4 plants effectively suppresses photorespiration, enhances photosynthetic efficiency, and optimizes water and nitrogen resource utilization.

The *NADP-ME* gene family exhibits limited membership across plant species, functionally diverging into photosynthetic and non-photosynthetic isoforms [9]. Photosynthetic NADP-ME operates in bundle sheath chloroplasts of C4 plants and in the cytosol of CAM plants, supplying ${\mathrm{CO}}_{2}$ for carbon fixation [8]. Conversely, non-photosynthetic NADP-ME functions in plastids or the cytosol, participating in diverse physiological processes such as cytosolic pH homeostasis, stomatal regulation, providing NADPH for biosynthesis (e.g., of flavonoids and lignin) and antioxidant defense (via the ascorbate–glutathione cycle), alongside plant stress responses [10,11,12,13]. Structurally, plant NADP-ME enzymes universally feature two conserved domains (N-terminal domain and NAD(P)-binding domain) and typically assemble into tetrameric complexes [14,15].

Beyond its role in C4 photosynthetic carbon fixation, NADP-ME has emerged as a critical regulator of plant abiotic stress responses [16]. Studies in multiple plant species have established a conserved regulatory pattern of *NADP-ME* under osmotic stress. Under drought and salinity stress, elevated NADP-ME activity accompanied by increased chloroplastic *NADP-ME* transcripts and decreased cytosolic *NADP-ME* expression was observed in tobacco (*Nicotiana tabacum*) following PEG treatment or drought exposure [17]. Conversely, Potato virus Y (PVY) infection selectively enhanced cytosolic *NADP-ME* transcription in tobacco, suggesting distinct regulatory mechanisms for biotic versus abiotic stress responses [18]. This subcellular-specific regulation implies functionally distinct roles of NADP-ME isoforms in stress adaptation. Functional validation studies have demonstrated the causal role of plastidic *NADP-ME* in drought tolerance: constitutive expression of maize (*Zea mays*) *ZmNADP-ME* in tobacco chloroplasts conferred drought resistance by reducing stomatal conductance and improving water use efficiency [19]. Similarly, salt stress induces NADP-ME activity in rice (*Oryza sativa*), and transgenic *Arabidopsis* (*Arabidopsis thaliana*) plants overexpressing *OsNADP-ME2*, *OsNADP-ME3*, or *OsNADP-ME4* exhibited enhanced osmotic regulation and improved salt tolerance [20,21,22]. Notably, overexpression of *OsNADP-ME4* conferred tolerance to both drought and salt stresses [20], highlighting the dual function of specific NADP-ME isoforms. Heterologous expression experiments have further confirmed the conserved stress tolerance function across diverse plant species: overexpression of sweet sorghum (*Sorghum bicolor*) *SbNADP-ME* in *Arabidopsis* elevated salt tolerance by enhancing osmotic regulation, 1,1-diphenyl-2-picrylhydrazyl (DPPH) radical scavenging capacity, and reducing membrane peroxidation [23], while *SaNADP-ME4* overexpression from the halophyte *Salsola laricifolia* simultaneously enhanced drought and salt stress tolerance in *Arabidopsis* [24]. Beyond osmotic stress, NADP-ME also participates in heavy metal detoxification, as demonstrated by the observation that overexpression of *Stylosanthes guianensis SgME1* increased aluminum tolerance in *Arabidopsis* by promoting malate synthesis and efflux [25].

In addition to osmotic and ionic stresses, NADP-ME is also involved in plant responses to temperature extremes, oxidative stress, and pathogen infection, primarily through modulating NADPH-dependent redox homeostasis. Low temperatures upregulated *NADP-ME* transcription in maize and sweet potato (*Dioscorea esculenta*) [26,27], suggesting its role in cold acclimation. UV radiation enhanced NADP-ME activity in *Egeria densa*, concomitant with increased activities of antioxidant enzymes including superoxide dismutase (SOD), peroxidase (POD), ascorbate peroxidase (APX), and catalase (CAT) [28], indicating a coordinated antioxidant defense response. The involvement of NADP-ME in plant immunity has also been established through genetic studies: *Arabidopsis nadp-me2* mutants exhibited weakened apoplastic ROS bursts upon pathogen challenge and increased susceptibility to *Colletotrichum higginsianum* [29,30], while *Magnaporthe oryzae* subverts rice immunity by suppressing OsNADP-ME2 and OsNADP-ME3 activity, thereby reducing cytosolic NADPH and ROS production [31]. These diverse stress responses converge on a common mechanism: NADP-ME provides NADPH for ROS scavenging and biosynthetic processes essential for stress adaptation. Despite these advances in understanding NADP-ME functions in C3 and C4 model plants, plastidic NADP-ME from C3-C4 intermediate species such as cassava remains poorly characterized under abiotic stress. The metabolic and structural characteristics of cassava are distinct from those of C3 and C4 plants, and further research will enhance understanding of their functions and regulatory mechanisms in plant stress resistance, providing a theoretical basis for the breeding of stress-tolerant cassava varieties.

In this study, we conducted a comprehensive analysis of *NADP-ME* genes in cassava, encompassing their characteristic features, phylogenetic relationships, gene duplication, and expression profiles across various tissues and organs. Additionally, comparative expression profiling of *NADP-ME* genes was performed at single-cell resolution using single-cell RNA sequencing (scRNA-seq) in cassava, *Arabidopsis*, and maize, species that represent different photosynthetic types. The expression patterns of *MeNADP-ME* genes under diverse abiotic stresses were further analyzed via quantitative real-time PCR (qRT-PCR). Laser scanning confocal microscopy revealed subcellular localization of MeNADP-ME3 to chloroplasts within cassava leaf protoplasts. Concurrently, functional characterization of *MeNADP-ME* was conducted through overexpression in *Arabidopsis*, investigating its role under abiotic stress conditions. This study will provide a foundation for research on *NADP-ME* genes in C3-C4 intermediate species and facilitate the breeding of stress-resistant cassava cultivars.

## 2. Materials and Methods

### 2.1. Genome-Wide Identification and Characterization of NADP-ME Gene Family

Genomic sequence data and annotation files for cassava were retrieved from the NCBI Genome Database. Full-length protein sequences were extracted from the genomic dataset using the annotation files. The hidden Markov model (HMM) profiles corresponding to the *NADP-ME* family (accession IDs: PF00390, PF03949 [32]) were downloaded from the Pfam database (Pfam 38.0). Using the hmmsearch function in HMMER 3.0 (European Bioinformatics Institute, Cambridge, UK), these NADP-ME HMM profiles were employed to query the cassava full-length protein sequences, with proteins meeting an E-value threshold of <$10^{-2}$ preliminarily retained. Concurrently, a BLASTp (https://blast.ncbi.nlm.nih.gov/Blast.cgi) search was performed against the cassava proteome using NADP-ME protein sequences from Arabidopsis as queries, selecting hits with E-value thresholds of <$10^{-2}$ [33,34]. Final identification of cassava *NADP-ME* family members was achieved by integrating these results with conserved domain verification via the CDD database, retaining candidates exhibiting >50% sequence similarity to reference *Arabidopsis* NADP-ME protein sequences. Physicochemical properties of the identified cassava NADP-ME proteins were predicted using ExPASy (https://web.expasy.org/protparam/).

Positional information of *MeNADP-ME* was retrieved from genome annotation files, with gene structures analyzed based on exon-intron organization. Conserved motifs in NADP-ME proteins from cassava, *Arabidopsis*, and maize were identified using MEME and functionally annotated via the InterPro database [32]. The upstream 2000-bp regions of coding sequences (CDS) from each *MeNADP-ME* family member were extracted, and cis-regulatory elements were identified using the PlantCARE database [35]. Data were classified, deduplicated, and visualized via the ggplot2 (Springer-Verlag, New York, NY, USA, v3.5.1) package in R (R Foundation for Statistical Computing, Vienna, Austria, v4.2.2).

### 2.2. Phylogenetic and Evolutionary Analysis

NADP-ME protein sequences from *Arabidopsis thaliana*, *Oryza sativa*, *Zea mays*, *Nicotiana tabacum*, *Flaveria pringlei*, *Populus trichocarpa*, *Sorghum bicolor*, *Aloe arborescens*, *Salsola laricifolia*, *Setaria italica*, *Triticum aestivum*, *Solanum lycopersicum*, *Ricinus communis*, and *Hevea brasiliensis* were aligned with cassava NADP-ME protein sequences using MUSCLE in MEGA 7.0 (Institute for Genomics and Evolutionary Medicine, Temple University, Philadelphia, PA, USA). A phylogenetic tree was constructed via the Neighbor-Joining method (Bootstrap replicates: 1000; partial deletion) [36]. Groups were delineated based on phylogenetic clades and visualized using iTOL (https://itol.embl.de/).

Synteny gene pairs within cassava and across species were identified and visualized using the One-Step MCScanX-SuperFast module in TBtools (v2.331). Nonsynonymous (Ka) and synonymous (Ks) substitution ratios for *MeNADP-ME* were computed with the Simple Ka/Ks Calculator [37]. Integrated visualization of all results was performed with TBtools (The Xia Rui Research Group of South Agricultural University, Guangzhou, China, v2.331) [37].

### 2.3. Functional Annotation and Enrichment Analysis

For functional enrichment analysis, MeNADP-ME protein sequences were annotated through the eggNOG-mapper platform for Gene Ontology (GO) and KEGG pathway enrichment [38]. Results were collated with TBtools, then integrated and visualized using ggplot2 (v3.5.1). Protein–protein interaction network for MeNADP-ME3 was predicted using the STRING database. Generated interaction networks were visualized using the tidygraph package (Chinese Software Developer Network, Beijing, China, v1.3.1) in R (v4.2.2). MeNADP-ME3 and its interacting proteins were annotated for KEGG pathway enrichment through the eggNOG-mapper platform [38].

### 2.4. Transcriptome Data Analysis

RNA sequencing (RNA-seq) data from diverse cassava tissues were downloaded from the ENA database (Project: PRJNA324539) [39]. Following data preprocessing to generate raw count matrices, transcripts per million (TPM) values were calculated for *MeNADP-ME* genes. After log2 transformation, results were visualized using TBtools to generate gene expression heatmaps [37].

For single-cell RNA sequencing (scRNA-seq) analysis, cassava leaf and storage root data (Projects: PRJCA019992, PRJNA895163) were analyzed [40,41]. Gene expression UMAP projections and bubble plots for cassava *NADP-ME* genes were generated via the Omicsmart platform. *Arabidopsis* leaf scRNA-seq data (GEO database: GSE161482) were processed using the Seurat package (New York Genome Center, New York, NY, USA, v4.3.0). Data standardization employed the SCTransform function, and mitochondrial genes were regressed out. Dimensionality reduction utilized >50 principal components (PCA) with a clustering resolution of 0.8. UMAP visualization applied PCA = 10, neighbors = 30, and mindistance = 0.1 [42]. Maize leaf scRNA-seq data (GEO database: GSE157759) were processed using the Seurat package (thresholds: percent.pt < 4; percent.mt < 0.75; nFeature_RNA > 1800 & < 7000). Standardization (SCTransform) and clustering (PCA = 50, resolution = 0.2) were performed.

### 2.5. Plant Materials and Growth Conditions

The cassava cultivar SC8, developed by the Institute of Biology at Hainan University, was used in this study. Healthy tissue culture seedlings grown on Murashige and Skoog (MS) medium for two months were selected for experimental treatments. Stress treatments included cold stress (4 °C incubation), heat stress (42 °C incubation), salt stress (uniform foliar application of 300 mM NaCl solution), and drought stress (20% *w/v* PEG-6000 solution). All solutions were sterilized by autoclaving prior to aseptic application in a laminar flow hood. The seedlings were treated with spraying for 48 h. Leaf samples were collected at 2 h, 4 h, 8 h, 12 h, 24 h, and 48 h post-treatment (three biological replicates), flash-frozen in liquid nitrogen, and stored at −80 °C for subsequent RNA extraction.

*Arabidopsis thaliana* ecotype Col-0 was used as the wild-type (WT) control. Plants were cultivated under controlled conditions: 60% relative humidity, 20–22 °C constant temperature, 16 h light/8 h dark photoperiod, and 200 μmol·$m^{-2}\cdot s^{-1}$ light intensity.

### 2.6. Molecular Cloning and Subcellular Localization

Total RNA was extracted from cassava leaves using the FastPure Plant Total RNA Isolation Kit (Vazyme Biomedical Technology, Nanjing, China; RC411). First-strand cDNA synthesis was performed with the HiScript III 1st Strand cDNA Synthesis Kit (Vazyme Biomedical Technology, Nanjing, China; R312). Primers for amplifying the *MeNADP-ME3* gene sequence (terminator-free) were designed via SnapGene (GSL Biotech, San Diego, CA, USA, v6.0.2) software based on restriction sites within the CaMV 35S promoter-driven pCAMBIA1300-eGFP vector: 1300-eGFPMeNADP-ME3-F(Sal I): GCGTCGACATGATCTCCTTGAAA, 1300-eGFPMeNADP-ME3-F(Kpn I): GGGGTACCCCGATAGCTTCGGTA. The MeNADP-ME3 fragment was amplified from cDNA templates using PrimeSTAR Max DNA Polymerase (Takara Biomedical Technology, Beijing, China; R045A). The resulting amplicon and pCAMBIA1300-eGFP vector were digested with Sal I and Kpn I, purified, ligated, and transformed to construct the recombinant pCAMBIA1300-MeNADP-ME3-eGFP expression vector.

For subcellular localization analysis, approximately 0.2 g of leaf tissue from cassava tissue culture seedlings (cv. SC8) was used for protoplast isolation according to the Plant Protoplasts Isolation Kit (Beyotime Biomedical Technology, Shanghai, China; C0362S). The isolated protoplasts were transfected with concentrated plasmid DNA using the Plant Protoplasts Transfection Kit (Beyotime Biomedical Technology, Shanghai, China; C0563S). Post-transfection, samples were cultured overnight under low light at room temperature. Transfected protoplasts were imaged using 488 nm excitation on Olympus FV3000 (Olympus Corporation, Haikou, China) laser-scanning confocal microscope.

### 2.7. Gene Expression Analysis by qRT-PCR

Gene-specific primers for *MeNADP-ME* were designed using NCBI Primer-BLAST (https://blast.ncbi.nlm.nih.gov/Blast.cgi) (Table S1). *β-tubulin* and *EF1α* served as reference genes. The amplification protocol comprised 40 cycles of: 95 °C for 5 s, 58 °C for 15 s, and 72 °C for 15 s (Table S2 for reaction mixture details). Three technical replicates were performed per sample. Relative gene expression was calculated using the $2^{-\Delta \Delta Ct}$ method [43]. Statistical analysis was conducted with SPSS (International Business Machines Corporation, Armonk, NY, USA, v23.0). Statistical significance was determined using Tukey’s test. Data visualization was performed using Origin (OriginLab, Northampton, MA, USA, 2018).

### 2.8. Generation and Functional Characterization of Transgenic Arabidopsis

The pCAMBIA1300-MeNADP-ME3-eGFP vector was introduced into wild-type *Arabidopsis thaliana* via the floral dip method [44]. Transgenic seeds were selected on half-strength Murashige and Skoog (1/2 MS) medium supplemented with 50 μg/mL hygromycin. Genomic DNA was extracted from leaves of putative transgenic seedlings using the Plant Genomic DNA Kit (Tiangen Biomedical Technology, Beijing, China; DP305). PCR verification was performed using vector-specific primers: 1300-F (CTCTAGATACATCACAATCA) and 1300-R (GCGGTTCACCAGGGTGTC). Transgene-positive plants were further screened to obtain homozygous lines (T3 generation). *MeNADP-ME3* transcript levels in homozygous lines were quantified by qRT-PCR using *Arabidopsis ACT2* and *TUB2* as reference genes. Primer sequences are listed in Table S3.

For stress tolerance assays, transgenic *Arabidopsis* seeds were vernalized and grown on standard 1/2 MS medium for 5 days. For heat stress treatment, seedlings were exposed to 42 °C for 1 h, then incubated vertically at 21 °C for 7 days prior to observation and imaging. Cold stress was applied at 4 °C for 1 h. For NaCl or PEG-induced osmotic stress, seedlings were transferred to 1/2 MS medium supplemented with 100 mM NaCl or 7% PEG, respectively, and cultured vertically at 21 °C for 7 days before imaging. Root length measurements were performed in biological triplicates.

For physiological parameter determination, seven-day-old WT and transgenic *Arabidopsis* seedlings were transplanted into 10 cm × 10 cm pots containing a nutrient soil/vermiculite mixture (2:1, *v/v*). Plants were grown for 21 days under normal conditions in a substrate consisting of peat moss (Pindstrup, 0–6 mm; pH-balanced, organic matter-rich) and vermiculite (supplemented with trace elements including Fe and Mg) prior to treatment. For salt stress, plants were irrigated with 300 mM NaCl for 3 days, while drought stress was imposed via natural dehydration for 7 days. Pre- and post-stress, 0.1 g rosette leaf samples per plant were collected to quantify MDA content, $H_{2}O_{2}$ levels, and activities of antioxidant enzymes (CAT, SOD, POD) using detection kits (Jiancheng Bioengineering Institute, Nanjing, China; A003, A064, A007, A001, A084). Statistical significance was determined using Tukey’s test. Data were analyzed statistically with SPSS (v23.0) and visualized using Origin (2018).

## 3. Results

### 3.1. Identification of Cassava NADP-ME Genes

A total of four *MeNADP-ME* genes were identified in the cassava genome through HMMER domain analysis and BLASTp searches. Table S4 summarizes the fundamental characteristics and physicochemical properties of these *MeNADP-ME* genes, including gene IDs, number of amino acids, molecular weight, theoretical isoelectric point, instability index, aliphatic index, and grand average of hydropathicity. Chromosomal localization analysis revealed that *MeNADP-ME1* and *MeNADP-ME4* reside on chromosome 16, while *MeNADP-ME2* and *MeNADP-ME3* are positioned on chromosomes 11 and 4, respectively (Table S5).

### 3.2. Phylogenetic Analysis of the NADP-ME Gene Family

**Figure 1:**
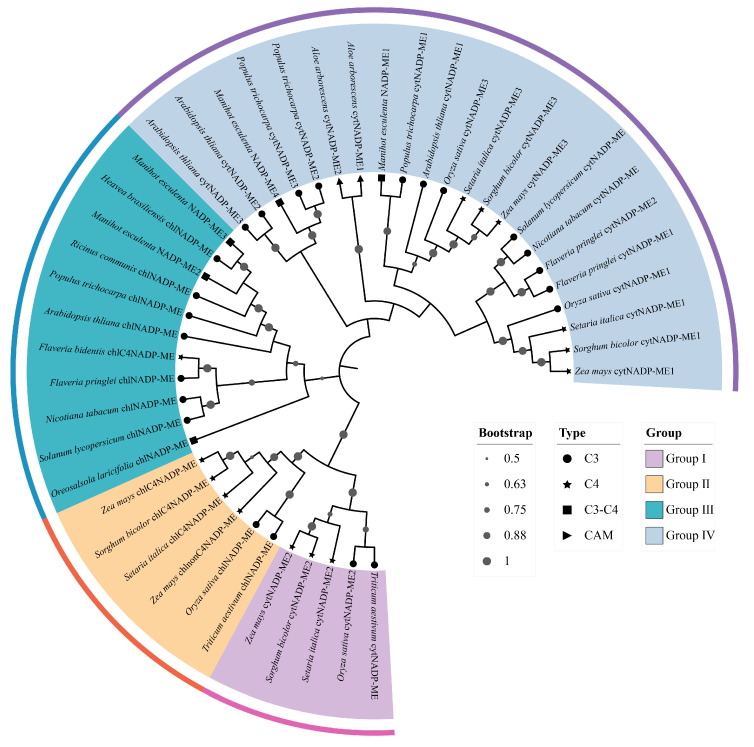
Phylogenetic trees of the NADP-ME family of plants of different photosynthetic types. Different colors represent various groups: Group I (cytosolic monocot types), Group II (plastidic monocot types), Group III (cytosolic eudicot types), and Group IV (cytosolic types shared by monocots and eudicots). Bootstrap values less than 0.5 were concealed.

Phylogenetic analysis of NADP-ME protein evolutionary relationships among cassava and other species was performed using the neighbor-joining method (Table S6). Based on branching patterns in the phylogenetic tree, plant NADP-MEs were classified into four subgroups [36]: Group I (cytosolic monocot types), Group II (plastidic monocot types), Group III (plastidic eudicot types), and Group IV (cytosolic types shared by monocots and eudicots) (Figure 1). Specifically, MeNADP-ME1 and MeNADP-ME4 formed a distinct clade within Group IV, whereas MeNADP-ME2 and MeNADP-ME3 clustered within Group III. The phylogenetic clustering patterns corresponded with evolutionary relationships and subcellular localization predictions, revealing higher homology between MeNADP-MEs and orthologs from rubber tree (*Hevea brasiliensis*) and castor bean (*Ricinus communis*), both members of the Euphorbiaceae family.

### 3.3. Gene Structure and Conserved Motif Analysis

**Figure 2:**
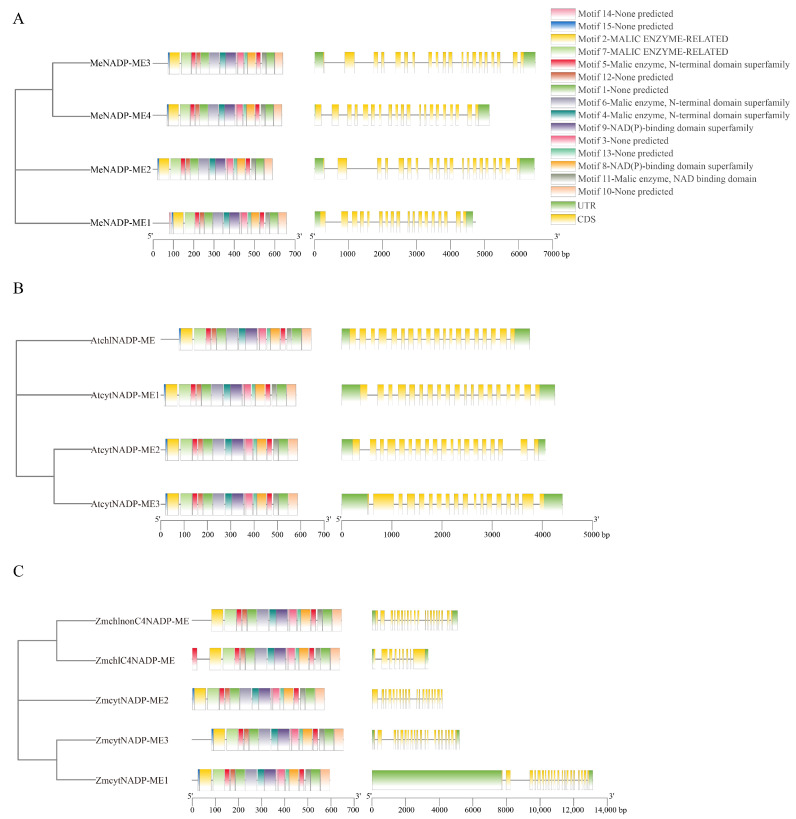
The conserved motifs and gene structures of the NADP-ME family. (**A**): Cassava; (**B**): *Arabidopsis*; (**C**): Maize.

Fifteen motifs were identified in the *NADP-ME* family of cassava, *Arabidopsis*, and maize (Table S7). Variations among the *NADP-ME* family members across the three species were principally localized to the N-terminal regions (Figure 2). Notably, MeNADP-ME1 possesses a unique N-terminal motif (Motif 14), ZmchlC4NADP-ME contains a distinctive N-terminal domain (Motif 5), while ZmchlnonC4NADP-ME lacked Motif 15 (a motif consistently conserved in other *NADP-ME* family members). The core functional domains, including the Malic enzyme N-terminal domain superfamily and NAD(P)-binding domain superfamily, were highly conserved across all family members. Analysis of the exon-intron structures of *NADP-ME* genes revealed that all members across the studied species contain introns, with the number of exons ranging from 12 to 15. *ZmcytNADP-ME1* exhibited the longest gene length and a unique gene structure with extended intron regions compared to other members.

### 3.4. Intragenomic and Interspecific Synteny Analysis

**Figure 3:**
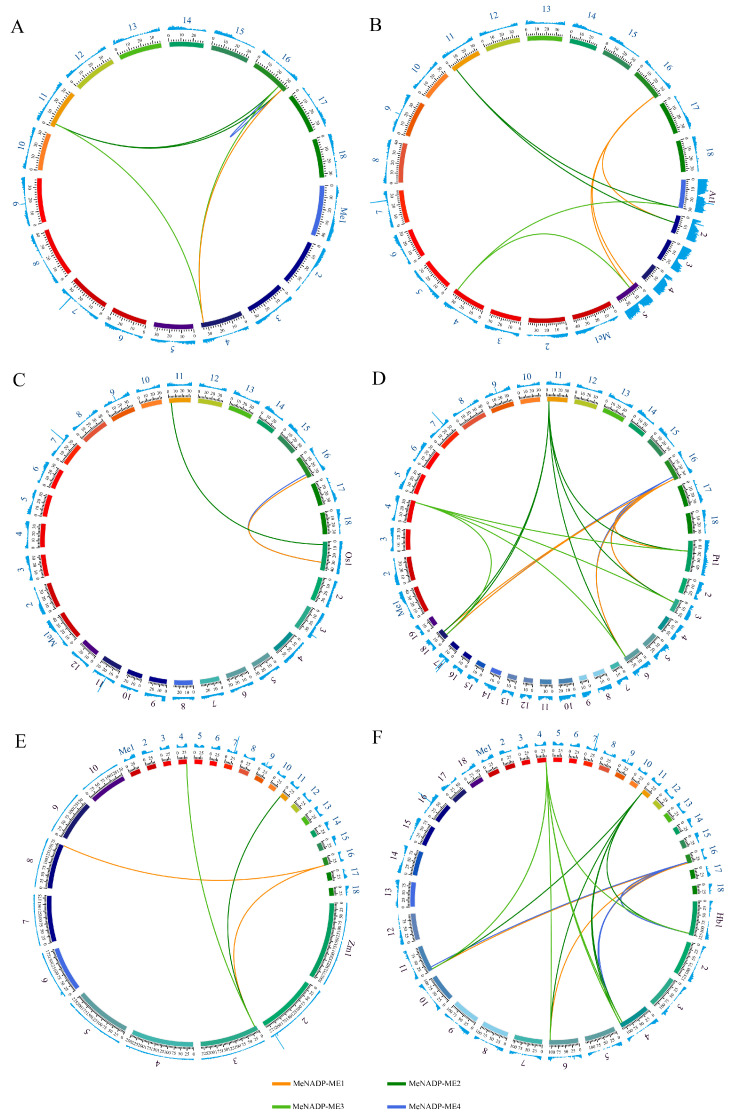
Syntenic analysis of the NADP-ME gene between cassava and species. (**A**): *Manihot esculenta* Crantz; (**B**): *Arabidopsis thaliana*; (**C**): *Oryza sativa*; (**D**): *Populus trichocarpa*; (**E**): *Zea mays*; (**F**): *Hevea brasiliensis*. The blue numbers represent the chromosome numbers of cassava, and the purple numbers correspond to those of other species.

Based on intraspecies synteny analysis of *MeNADP-ME* genes, six pairs of segmentally duplicated genes were identified (Figure 3A), including *MeNADP-ME1* with *MeNADP-ME2*, *MeNADP-ME3*, and *MeNADP-ME4*; *MeNADP-ME2* with *MeNADP-ME3* and *MeNADP-ME4*; and *MeNADP-ME3* with *MeNADP-ME4*. Gene and genome duplication represents a major evolutionary force driving biological diversity and complexity, and the results of intra-species synteny analysis indicate that segmental duplication serves as the primary driver of *MeNADP-ME* gene evolution. Ka/Ks ratios (nonsynonymous to synonymous substitution rates) were calculated for the identified duplicated gene pairs to infer selective pressures (Table S8). Analysis revealed that the duplicate gene pair *MeNADP-ME3*/*MeNADP-ME4* (Ka/Ks > 1) arose under positive selection pressure, while other gene pairs exhibited Ka/Ks ratios less than 1, indicating purifying selection. To further elucidate the evolutionary relationships within the *NADP-ME* family, syntenic genes between cassava and plant species exhibiting different photosynthetic types were analyzed (Figure 3B–F, Table S9). The highest numbers of syntenic genes were detected between cassava and rubber tree (18 pairs) as well as the dicotyledonous plant *Populus trichocarpa* (18 pairs). Seven and three pairs of syntenic genes were detected with the model plants *Arabidopsis* and rice, respectively. Four pairs of syntenic genes were identified with the monocot C4 plant maize. These findings further elucidate the evolutionary origins and conservation patterns of *MeNADP-ME* genes across diverse plant lineages.

### 3.5. Cis-Regulatory Elements Analysis and GO/KEGG Enrichment Analysis

**Figure 4:**
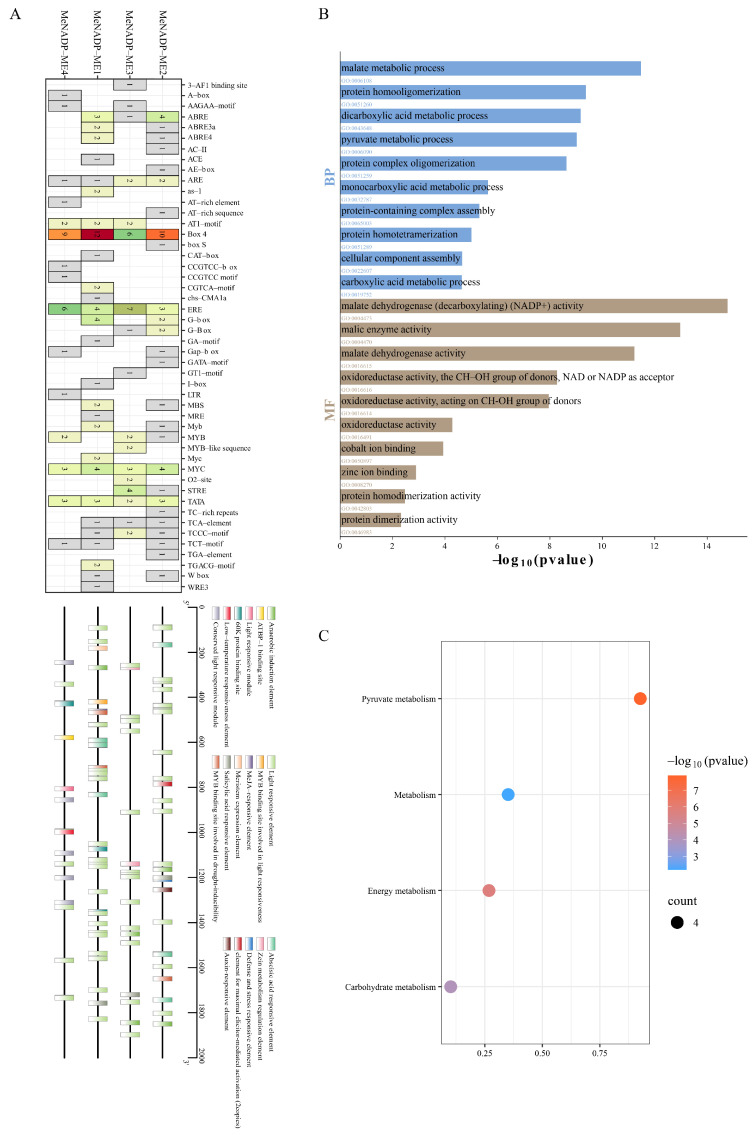
Cis-regulatory elements analysis and GO/KEGG enrichment analysis of *MeNADP-ME*. (**A**): Analysis of cis-acting elements of *MeNADP-ME*; (**B**): GO enrichment analysis of *MeNADP-ME* (Top 10); (**C**): KEGG enrichment analysis of *MeNADP-ME* (Top 5).

Seventeen distinct types of cis-acting regulatory elements were identified in the promoter regions of *MeNADP-ME* genes (Figure 4A, Table S10). Within the *MeNADP-ME* family, all sequences harbor a substantial quantity of light-responsive elements, particularly the Box 4 element, which is highly enriched in every gene (ranging from 6 to 13 instances per promoter), followed by G-box and GT1-motif elements. Regarding hormone responses, the *MeNADP-ME* family includes various hormone-responsive elements, such as methyl jasmonate (MeJA)-responsive CGTCA-motif and TGACG-motif, abscisic acid (ABA)-responsive ABRE elements, and ethylene-responsive ERE elements. The *MeNADP-ME* family also contains elements associated with diverse stress responses, including anaerobic induction (ARE), low-temperature responsiveness (LTR), drought inducibility (MBS), and defense/stress responsiveness (TC-rich repeats). Additionally, the presence of a CAT-box meristem expression element within *MeNADP-ME1* suggests its potential involvement in actively proliferating meristematic tissues, linking development to stress responses. Furthermore, all *MeNADP-ME* family members contain MYB- and MYC-binding sites involved in transcriptional regulation. These findings demonstrate that the *MeNADP-ME* genes play important roles in plant adaptation to environmental shifts, stress responses, and the coordination of growth and development.

Based on screening and collation, the top ten most enriched GO terms and five enriched KEGG pathways were identified (Figure 4B,C). GO enrichment analysis revealed that the *MeNADP-ME* family is predominantly associated with malate metabolic process and pyruvate metabolic process under biological processes (BP) category. In the molecular function (MF) category, these proteins primarily function through protein homooligomerization, malic enzyme activity, and malate dehydrogenase (decarboxylating) (NADP+) activity. KEGG enrichment results indicated that the *MeNADP-ME* family is significantly enriched in core metabolic pathways related to photosynthetic carbon fixation, including pyruvate metabolism, carbohydrate metabolism, energy metabolism, and carbon metabolism. The high enrichment in these pathways underscores the central role of NADP-ME in primary metabolism and photosynthetic processes.

### 3.6. RNA-Seq Analysis of MeNADP-ME Genes

**Figure 5:**
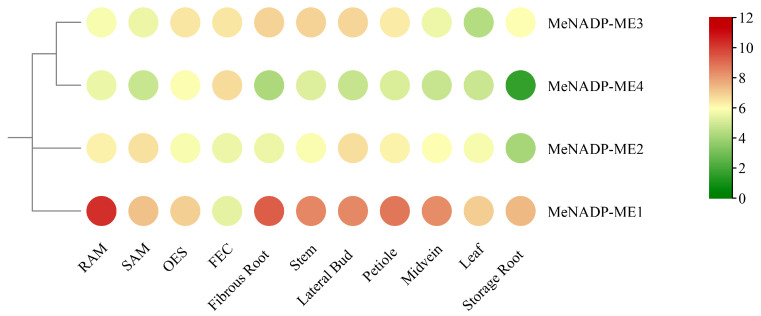
The expression of *MeNADP-ME* in different tissues and organs.

Through analysis of raw transcriptomic data downloaded from the ENA database (PRJNA324539) for 11 cassava tissues and organs, the expression patterns of *MeNADP-ME* genes across different tissues were characterized, and expression profiles were generated (Figure 5, Table S11). *MeNADP-ME1* was highly expressed in all tissues except friable embryogenic callus (FEC), with the highest expression observed in the root apical meristem (RAM), followed by stems and fibrous roots—consistent with the predicted expression pattern based on prior cis-regulatory element analysis, particularly the presence of CAT-box element. *MeNADP-ME2* exhibited low expression across most tissues and organs but showed elevated expression specifically in the shoot apical meristem (SAM) and lateral buds, suggesting its specialized role in shoot development. *MeNADP-ME3* displayed relatively high and constitutive expression levels across multiple tissues and organs, especially in stems, lateral buds, and leaves, indicating its housekeeping function in cassava. *MeNADP-ME4* was highly expressed in FEC and ovular embryogenic suspensor (OES) tissues, while expression remained low in other mature organs, suggesting its specific involvement in early embryonic development. These distinct and tissue-specific expression patterns suggest that *MeNADP-ME* genes have undergone functional divergence and play pivotal roles in cassava growth and development, warranting further investigation.

### 3.7. ScRNA-Seq Analysis of NADP-ME Genes in Cassava, Arabidopsis, and Maize

**Figure 6:**
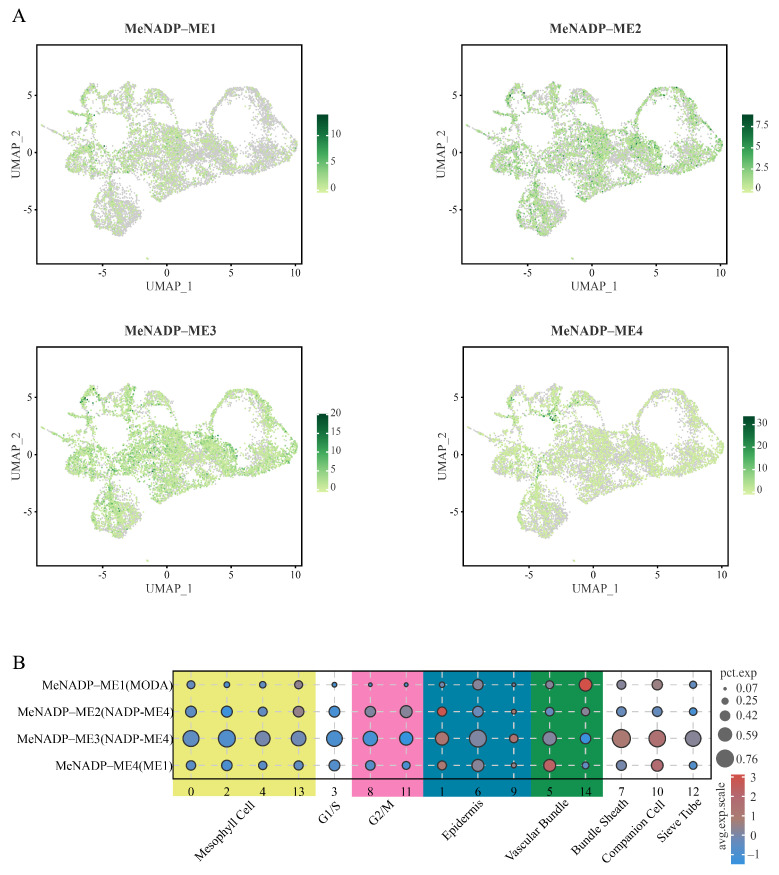
Single-cell transcriptome analysis of *MeNADP-ME* in cassava leaves. (**A**): UMAP map of *MeNADP-ME*; (**B**): Bubble chart of *MeNADP-ME* expression.

**Figure 7:**
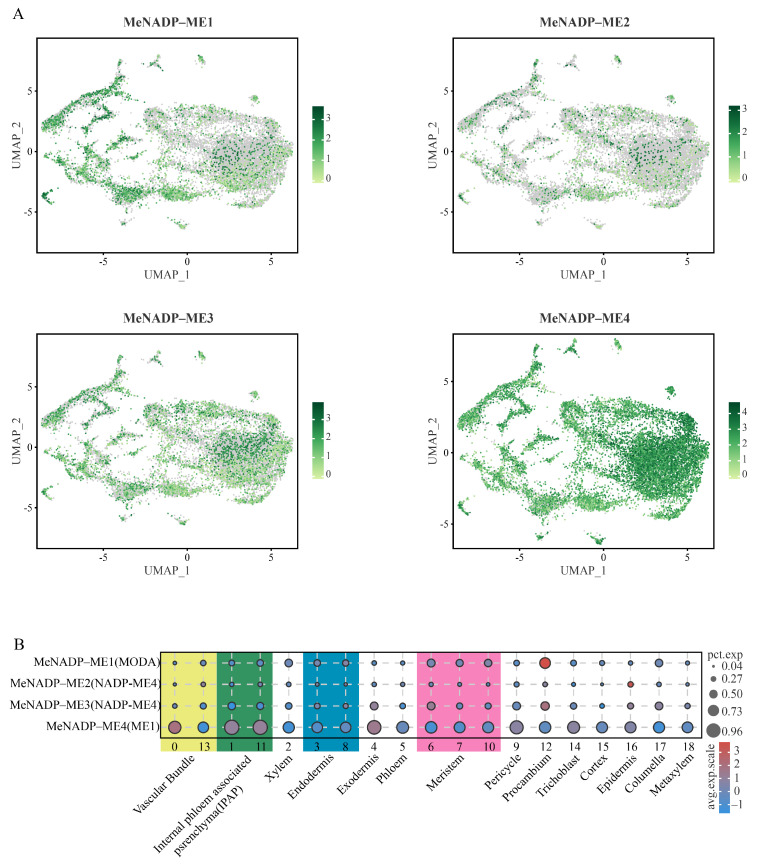
Single-cell transcriptome analysis of *MeNADP-ME* in cassava tuberous roots. (**A**): UMAP map of *MeNADP-ME*; (**B**): Bubble chart of *MeNADP-ME* expression.

Based on single-cell RNA sequencing analyses of cassava leaves and tuberous roots (PRJCA019992, PRJNA895163), the expression patterns of *MeNADP-ME* genes were characterized at single-cell resolution (Figure 6 and Figure 7; Tables S12 and S13). In leaves (Figure 6), *MeNADP-ME1* exhibited high expression restricted to vascular cells, albeit with relatively low cellular abundance. *MeNADP-ME2* was exclusively highly expressed in a limited number of epidermal cells, showing extremely low cellular abundance across all cell types. *MeNADP-ME3* showed the most prominent expression pattern, with elevated expression in bundle sheath cells and companion cells, and particularly high cellular abundance observed in bundle sheath cells, suggesting its critical role in vascular-associated metabolism. *MeNADP-ME4* displayed moderate to high expression levels across multiple cell types including vascular cells, epidermal cells, and companion cells, though its cellular abundance remained relatively low in all these cell types. In tuberous roots (Figure 7), *MeNADP-ME1* demonstrated prominent expression in numerous procambial cells. *MeNADP-ME2* maintained high expression exclusively within sparse epidermal cells, consistent with its leaf expression pattern. In contrast, *MeNADP-ME3* showed consistently low expression across diverse root cell types, markedly different from its high expression in leaves. Furthermore, *MeNADP-ME4* exhibited the highest expression levels in vascular bundle cells, phloem parenchyma cells, and exodermis cells, accompanied by high cellular abundance within these populations, indicating its important role in root storage organ development and nutrient transport.

**Figure 8:**
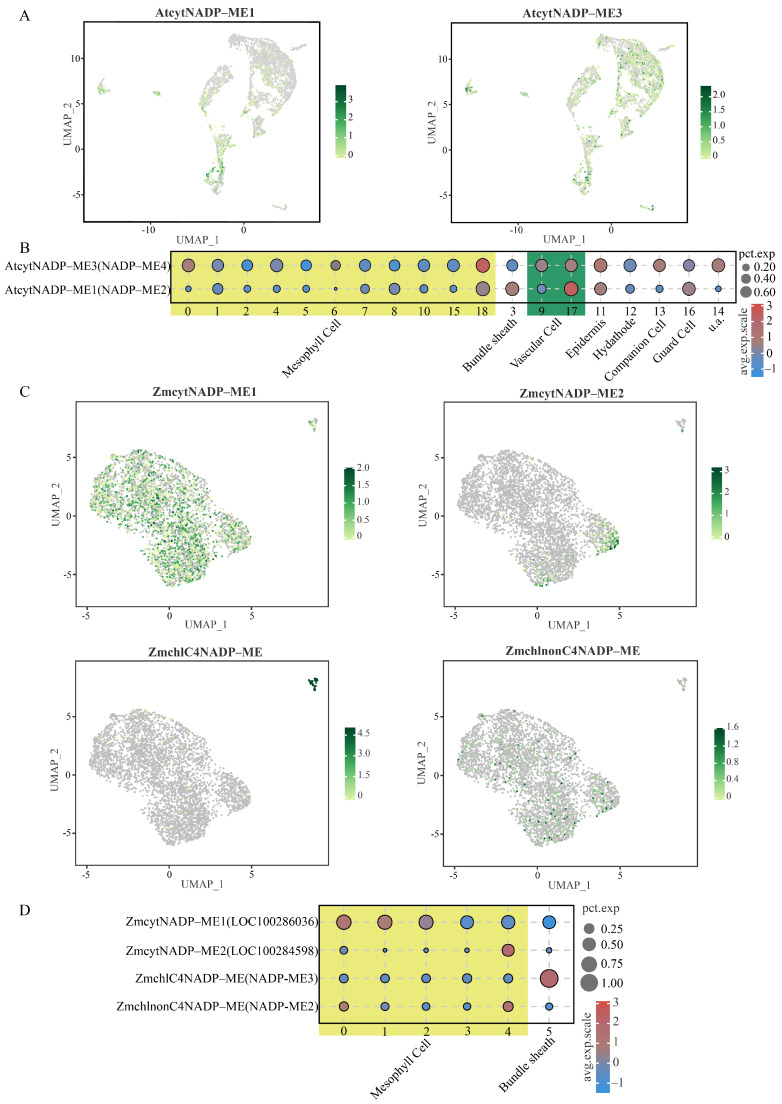
Single-cell transcriptome analysis of *NADP-ME* genes in *Arabidopsis* and maize leaves. (**A**): UMAP map of *AtNADP-ME*; (**B**): Bubble chart of *AtNADP-ME* expression; (**C**): UMAP map of *ZmNADP-ME*; (**D**): Bubble chart of *ZmNADP-ME* expression.

To investigate the expression patterns of *NADP-ME* genes across different photosynthetic plant types at single-cell resolution, we analyzed single-cell transcriptomic datasets of *Arabidopsis* and maize leaves (GSE161482, GSE157759; Figure 8, Tables S14 and S15). In *Arabidopsis* leaves (Figure 8A,B), expression was detected for only two *NADP-ME* genes: *AtcytNADP-ME1* (corresponding to NADP-ME4 nomenclature) exhibited high expression in vascular cells, with moderate expression levels additionally observed in guard cells, epidermal cells, and bundle sheath cells. *AtcytNADP-ME3* (corresponding to NADP-ME3 nomenclature) displayed a diffuse expression pattern across multiple cell types, showing higher expression in mesophyll cells and epidermal cells. In maize leaves (Figure 8C,D), four *NADP-ME* genes were expressed. Contrasting with the *Arabidopsis* expression patterns, *ZmcytNADP-ME1* (LOC100286036) and *ZmcytNADP-ME2* (LOC100284598) were highly expressed in mesophyll cells, although *ZmcytNADP-ME2* expressing cells showed low cellular abundance. Notably, *ZmchlC4NADP-ME* (NADP-ME3) was highly expressed in bundle sheath cells but minimally expressed in mesophyll cells, paralleling the expression pattern of *MeNADP-ME3*. Furthermore, *ZmchlnonC4NADP-ME* (NADP-ME2) showed significant expression in mesophyll cells, albeit within a cellular population of low abundance. These comparative expression patterns across C3 (*Arabidopsis*), C3-C4 intermediate (cassava), and C4 (maize) species provide critical insights into the evolutionary trajectory of photosynthetic NADP-ME function.

**Figure 9:**
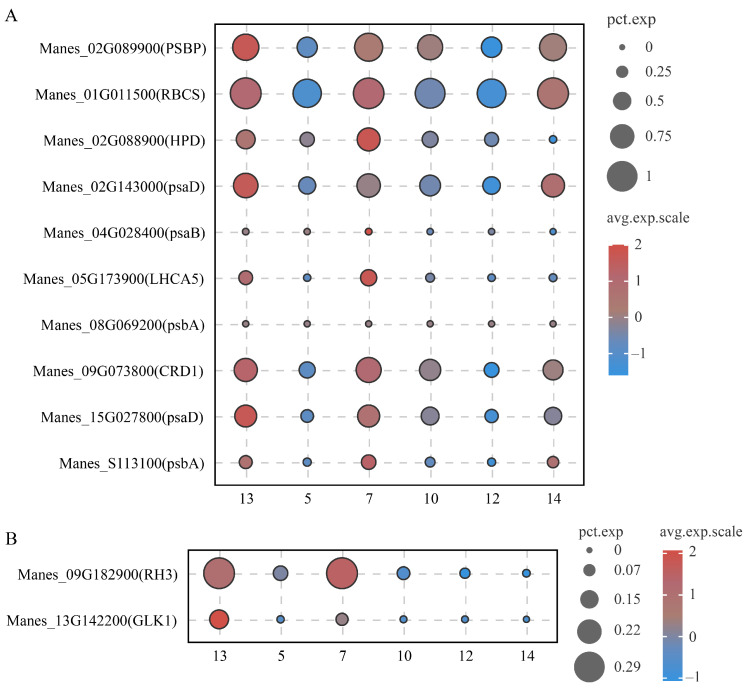
Single-cell transcriptome analysis of photosynthetically related genes and PD-related genes in typical cell subsets of cassava leaves. (**A**): Bubble chart of expression of photosynthetically related genes; (**B**): Bubble chart of expression of PD-related genes.

**Figure 10:**
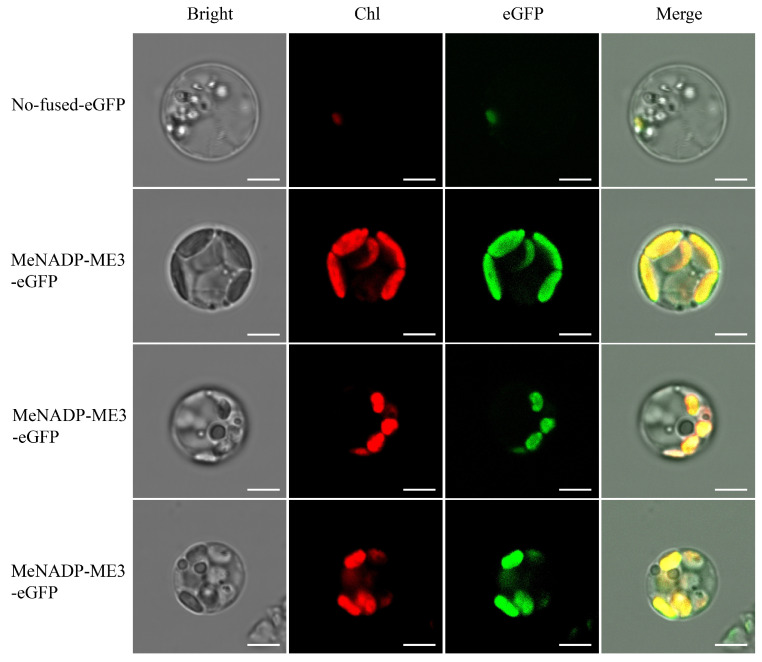
Subcellular localization of MeNADP-ME3 protein in cassava leaf protoplasts. Scale length (5 μm).

*MeNADP-ME3* exhibits an expression pattern at the single-cell level similar to that of *ZmchlC4NADP-ME* from the C4 plant maize, suggesting the potential existence of a limited C4 photosynthetic pathway in cassava. To further investigate this hypothesis, we analyzed the single-cell level expression profiles of photosynthesis-associated genes and PD-related genes in mesophyll cell subset 13 (adjacent to bundle sheath cells) and vascular tissue cell subsets (5, 7, 10, 12, 14) [40,45,46,47,48,49]. These findings (Figure 9) demonstrate that photosynthetic genes, including PHOSPHOENOLPYRUVATE CARBOXYLASE (PEPC, Manes_02G089900), RIBULOSE BISPHOSPHATE CARBOXYLASE SMALL CHAIN (RBCS, Manes_01G011500), HYDROXYPYRUVATE DECARBOXYLASE (HPD, Manes_02G088900), PLASMODESMATA-RELATED GENES (psaD, Manes_02G143000; psaB, Manes_04G028400), LIGHT-HARVESTING CHLOROPHYLL A/B BINDING PROTEIN 5 (LHCA5, Manes_05G173900), CHLOROPLAST RNA BINDING PROTEIN (CRD1, Manes_09G073800), and other photosynthesis-related genes (psaA, psaB, psbA), were highly co-expressed in mesophyll subset 13 and bundle sheath cells (subset 7), with substantial cellular abundance. Similarly, PD-related genes, including RH3 (Manes_09G182900) and GLK1 (Manes_13G142200), exhibited elevated expression in these cell subsets, particularly in mesophyll subset 13 (adjacent to bundle sheath) and bundle sheath cells (subset 7). This distinctive co-expression pattern of photosynthetic genes and PD-related genes in adjacent mesophyll and bundle sheath cells provides strong molecular evidence supporting the existence of a limited C4 photosynthetic mechanism in cassava, potentially facilitating intercellular ${\mathrm{CO}}_{2}$ transport and concentration. Transient expression of the CaMV 35S promoter-driven pCAMBIA1300-MeNADP-ME3-eGFP construct in cassava leaf protoplasts revealed chloroplast localization of MeNADP-ME3 via confocal microscopy (Figure 10). The green fluorescence signal of MeNADP-ME3-eGFP fusion protein co-localized with the red chlorophyll autofluorescence, as evidenced by the yellow merged signal in the overlay images, while the no-fused-eGFP control showed diffuse cell nucleus distribution. This chloroplast localization pattern corroborates the potential role of MeNADP-ME3 in photosynthetic carbon metabolism and supports its functional similarity to C4-type NADP-ME in cassava.

### 3.8. Protein Interaction Prediction and KEGG Enrichment Analysis for MeNADP-ME3

**Figure 11:**
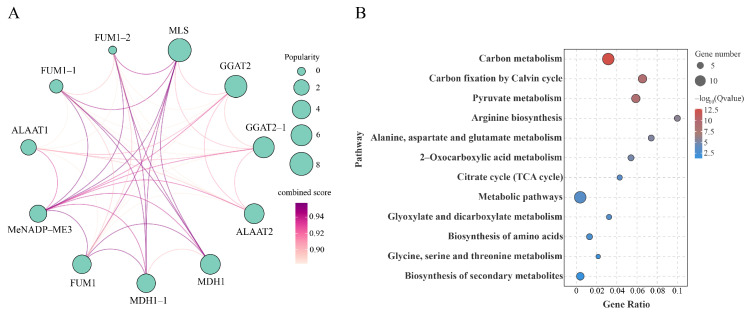
Prediction of MeNADP-ME3 protein–protein interaction and KEGG enrichment analysis. (**A**): Protein–protein interaction network; (**B**): KEGG enrichment analysis.

Based on protein–protein interaction network analysis of MeNADP-ME3 constructed using the STRING database (Figure 11A, Table S16), KEGG enrichment annotation revealed functional specializations of key interactors: Fumarate hydratase 1 and 2 (FUM1, FUM1-2) primarily engage in energy metabolism via the TCA cycle; Alanine aminotransferase 1 and 2 (ALAAT1, ALAAT2) predominantly participate in amino acid biosynthesis and interconversion; Malate dehydrogenase 1 and 1-1 (MDH1, MDH1-1) contribute to photosynthetic carbon assimilation and redox homeostasis regulation; Glutamate glyoxylate aminotransferase 2 and 2-1 (GGAT2, GGAT2-1) are mainly involved in photorespiration and amino acid metabolism; while Malate synthase (MLS) operates within the glyoxylate cycle (Figure 11B, Table S17). The interaction network showed strong connectivity between MeNADP-ME3 and its partners, with MLS exhibiting the highest interaction score, indicating a central metabolic hub. MeNADP-ME3 and its interacting partners were jointly enriched in carbon metabolism (highest significance), carbon fixation by Calvin cycle, pyruvate metabolism, and arginine biosynthesis pathways. Notably, the high enrichment in carbon metabolism and the strong interaction with MLS suggest that the low photorespiration characteristic of cassava may be attributed to the coordinated regulation between NADP-ME-mediated malate decarboxylation and the glyoxylate cycle, potentially providing an alternative pathway to bypass photorespiratory ${\mathrm{CO}}_{2}$ loss.

### 3.9. Expression Profiles of MeNADP-ME Under Different Abiotic Stresses

**Figure 12:**
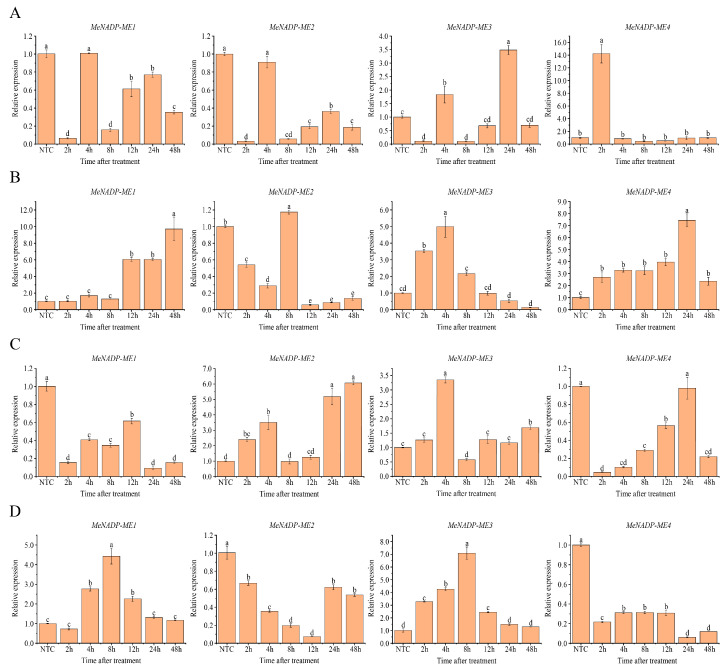
Expression profiles of *MeNADP-ME* under different abiotic stresses. (**A**): Cold stress (4 °C); (**B**): Heat stress (42 °C); (**C**): Salt stress (300 mM NaCl); (**D**): Drought stress (20% PEG). These values represent the average ± SE from three independent experiments. Different letter designations indicate significant differences (p < 0.05).

To investigate the expression patterns of *MeNADP-ME* in response to various abiotic stresses, qRT-PCR analysis was performed on leaves of cassava plantlets subjected to different stress treatments. Under 4 °C cold stress treatment (Figure 12A), *MeNADP-ME1* expression was significantly downregulated at 2 h post-treatment. Although it recovered to baseline levels at 4 h, it was significantly downregulated again at 8 h and remained suppressed through 48 h. Similarly, *MeNADP-ME2* expression decreased transiently at 2 h before returning to control levels, but exhibited significant downregulation at 8 h and remained low thereafter. *MeNADP-ME3* expression was transiently suppressed at 2 h, followed by significant upregulation at 4 h, before rapidly declining. However, it showed significant upregulation again at 24 h post-treatment, reaching its peak expression. In contrast, *MeNADP-ME4* expression was significantly and dramatically upregulated, peaking at 2 h, followed by a rapid decline to baseline levels by 8 h. Following 42 °C heat stress (Figure 12B), *MeNADP-ME1* expression was significantly upregulated starting at 12 h and reached its peak at 48 h. *MeNADP-ME2* expression initially declined transiently at 2 h, was significantly upregulated at 8 h, and then rapidly decreased to significantly lower levels at 24 h. *MeNADP-ME3* expression was rapidly and significantly induced, peaking at 4 h, before gradually declining over time. *MeNADP-ME4* expression was significantly upregulated after heat treatment, peaking at 24 h, displaying a trend similar to that of *MeNADP-ME1*. The distinct biphasic or sustained expression patterns of the four *MeNADP-ME* genes under cold and heat stresses suggest functional specialization: certain genes (*MeNADP-ME3*, *MeNADP-ME4*) may be associated with rapid cold/heat shock response, while others (*MeNADP-ME1*) may function in long-term temperature acclimation. This divergence reflects the differential regulatory strategies employed by plants to adapt and respond to thermal fluctuations and diverse environmental challenges.

Following salt stress treatment (300 mM NaCl) (Figure 12C), all *MeNADP-MEs* exhibited pronounced responses with distinct temporal dynamics. The expression levels of *MeNADP-ME1* and *MeNADP-ME4* were immediately and significantly downregulated at 2 h, though both showed gradual recovery trends over time. Notably, *MeNADP-ME4* rebounded to baseline levels at 24 h post-treatment before rapidly declining again at 48 h. In contrast, *MeNADP-ME2* and *MeNADP-ME3* were significantly upregulated immediately after salt treatment, with *MeNADP-ME3* reaching peak expression at 2 h, but both dropped sharply thereafter. *MeNADP-ME2* subsequently exhibited a secondary upregulation, peaking at 48 h, suggesting a potential role in long-term salt adaptation. Under drought stress simulated by 20% PEG (Figure 12D), *MeNADP-ME1* and *MeNADP-ME3* were markedly induced at 2 h but declined significantly to basal levels after 8 h of treatment, indicating an early rapid response mechanism. Conversely, *MeNADP-ME2* and *MeNADP-ME4* displayed downregulation trends throughout the treatment period. Given that both salt and drought stresses cause osmotic imbalance and oxidative damage in plants, the divergent and temporally distinct response patterns of these four *MeNADP-ME* genes suggest stress-specific regulatory mechanisms and functional specialization underlying plant adaptation to distinct environmental challenges. The rapid induction of *MeNADP-ME3* under both salt (early phase) and drought stresses further supports its critical role in osmotic stress responses.

### 3.10. Overexpression of MeNADP-ME3 Confers Enhanced Tolerance to Salt and Drought Stresses in Arabidopsis

**Figure 13:**
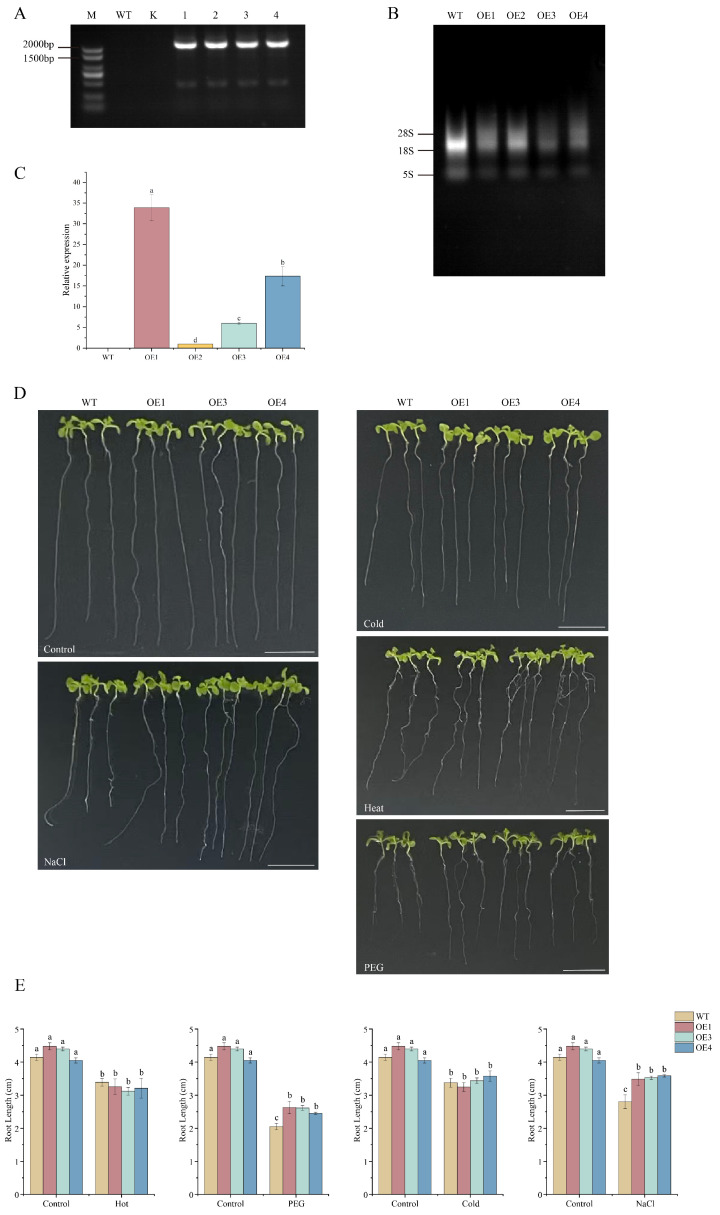
Generation of *MeNADP-ME3* transgenic *Arabidopsis* and the effects of different stress treatments on the root length. (**A**): DNA molecular identification of transgenic *Arabidopsis*. M: 2000 bp DNA Marker, WT: Wild type, K: Blank control; (**B**): Total RNA in *Arabidopsis* leaves; (**C**): The relative expression level of *MeNADP-ME3* transgenic *Arabidopsis*. (**D**): The growth state of the initial roots under stress treatment, the length of the ruler is 1 cm; (**E**): Determination of root length. These values represent the average ± SE from three independent experiments. Different letter designations indicate significant differences (p < 0.05).

Transgenic *Arabidopsis* plants overexpressing *MeNADP-ME3* were generated via the floral dip method. Four independent transgenic lines (OE1-4) were obtained following screening on hygromycin-containing medium (Figure 13A). PCR amplification with vector-specific primers confirmed the presence of the transgene in all four lines, while no amplification was observed in wild-type (WT) or blank control (K). Total RNA integrity was verified by agarose gel electrophoresis (Figure 13B), showing clear ribosomal RNA bands. RT-qPCR analysis confirmed *MeNADP-ME3* expression levels in transgenic lines (Figure 13C), with OE1 showing the highest expression, followed by OE3 and OE4. Three lines (OE1, OE3, and OE4) with the highest and most consistent relative expression were selected for subsequent functional experiments. As shown in Figure 13D,E, no significant difference in primary root length was observed between WT and transgenic seedlings under control conditions, confirming that *MeNADP-ME3* overexpression does not affect normal development. Following exposure to various stresses, transgenic lines exhibited significantly longer primary roots than WT under PEG-mediated drought stress and NaCl salt stress, representing approximately 60% and 40% improvements, respectively. Conversely, temperature stresses (cold at 4 °C for 1 h/heat at 42 °C for 1 h) produced no distinct difference in root elongation between genotypes, with both showing similar root lengths. These findings indicate that *MeNADP-ME3* overexpression specifically enhances tolerance to osmotic and ionic stressors but does not alter temperature stress responses in *Arabidopsis*, suggesting its primary function in maintaining cellular homeostasis under water-deficit conditions.

**Figure 14:**
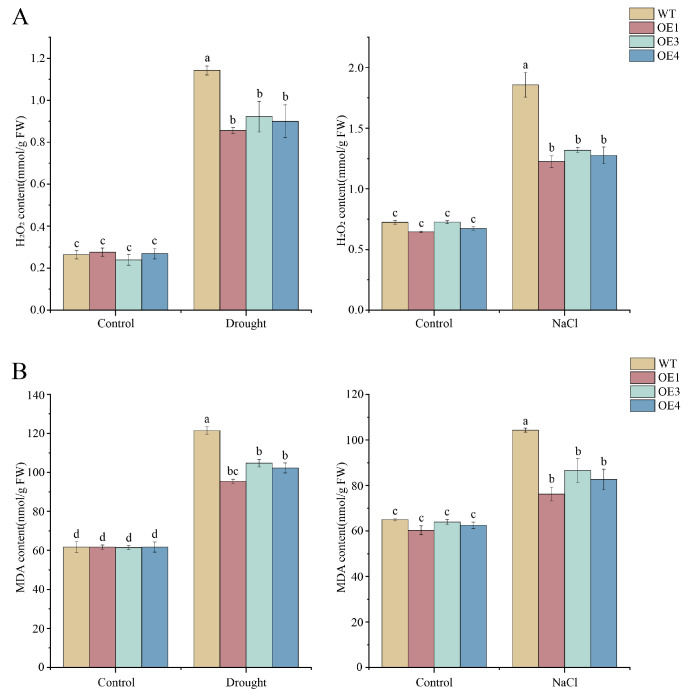

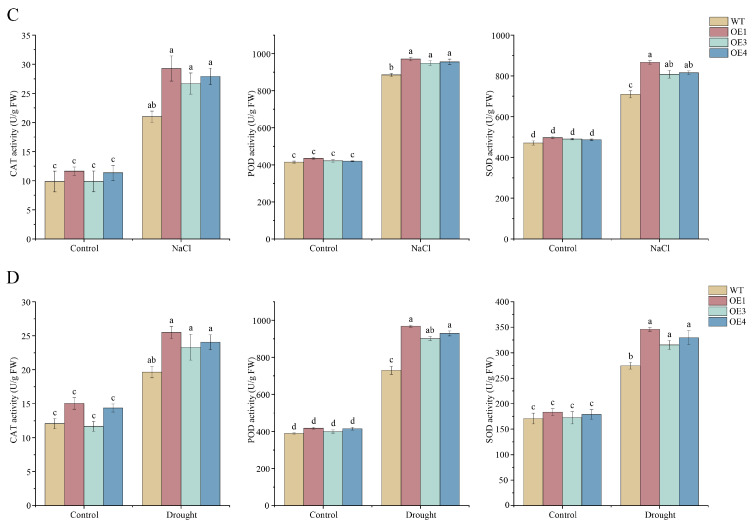
Determination of stress-resistance parameters in transgenic *Arabidopsis* under drought and salt stress. (**A**): Determination of $H_{2}O_{2}$ content under salt stress and drought stress; (**B**): Determination of MDA content under salt stress and drought stress; (**C**): Determination of antioxidant enzyme activities under salt stress; (**D**): Determination of antioxidant enzyme activities under drought stress. These values represent the average ± SE from three independent experiments. Different letter designations indicate significant differences (p < 0.05).

Following 21-day growth under soil culture conditions, stress tolerance-related parameters were measured in WT and transgenic plants subsequent to salt and drought stress treatments (Figure 14). Malondialdehyde (MDA) and $H_{2}O_{2}$ contents are established indicators for evaluating membrane lipid peroxidation and oxidative stress status, respectively. Under control conditions, basal MDA levels and $H_{2}O_{2}$ contents were comparable between WT and transgenic lines. After salt stress (300 mM NaCl for 3 days), both MDA and $H_{2}O_{2}$ levels increased significantly in all plants; however, transgenic lines exhibited significantly lower accumulation compared to WT (Figure 14A,B). Specifically, WT plants showed elevated MDA content and $H_{2}O_{2}$ levels under salt stress, while transgenic lines maintained significantly lower levels, representing approximately 27% and 32% reductions, respectively. Similar patterns were observed under drought stress (natural dehydration for 7 days), with transgenic lines showing approximately 20–25% lower MDA and $H_{2}O_{2}$ accumulation compared to WT.

Activities of antioxidant enzymes (CAT, SOD, POD) constitute additional critical metrics in stress response studies, as they catalyze the detoxification of reactive oxygen species (ROS). As shown in Figure 14C,D, no significant differences in these enzyme activities were detected between WT and transgenic plants under control conditions. In contrast, under salt stress, all transgenic Arabidopsis lines displayed significantly higher antioxidant enzyme activities than WT: CAT activity increased by approximately 30%, SOD activity by approximately 75%, and POD activity by approximately 120%. Under drought stress, similar enhancement patterns were observed, with OE lines showing 20–25% higher CAT, 70–80% higher SOD, and 80–90% higher POD activities compared to WT. Collectively, these results demonstrate that overexpression of *MeNADP-ME3* enhances salt and drought tolerance in transgenic *Arabidopsis* by augmenting antioxidant enzyme capacity, thereby reducing oxidative damage and maintaining cellular redox homeostasis under stress conditions.

## 4. Discussion

NADP-ME is ubiquitously distributed across diverse photosynthetic lineages, encompassing algae, mosses, ferns, and seed plants [50]. The majority of C4 species belong to monocotyledons, particularly within the Poaceae (4600 species) and Cyperaceae (1600 species). In contrast, only approximately 1600 C4 species have been identified in dicotyledons, distributed across 16 families, with nearly 75% concentrated within four dominant families: Amaranthaceae, Euphorbiaceae, Asteraceae, and Caryophyllaceae [51]. In this study, four *MeNADP-ME* genes were identified in the cassava genome through integrated HMM domain analysis and BLASTp homology searches. Evolutionary analyses revealed that although the cassava genome (640 Mb) is nearly twice the size of the rice genome (386 Mb), both possess an identical number of *NADP-ME* genes. This finding also supports the notion that expansion of the *NADP-ME* gene family did not result from whole-genome duplication events, but rather from lineage-specific expansion patterns following the divergence of monocotyledons and dicotyledons [36,52].

Phylogenetic analysis provides critical insights into evolutionary relationships among species, genes, or populations. In this study, phylogenetic analysis was conducted to investigate evolutionary relationships among NADP-ME proteins from cassava and diverse plant lineages with varying photosynthetic types. These NADP-MEs were categorized into four clades: Group I (cytosolic monocot types), Group II (plastidic monocot types), Group III (cytosolic eudicot types), and Group IV (cytosolic types shared by monocots and eudicots) [36]. Phylogenetic clustering aligned with evolutionary relationships and subcellular localization. Cassava displays closer phylogenetic affinity to its congeneric Euphorbiaceae species rubber tree and castor bean, as well as to *Arabidopsis*, *Populus trichocarpa*, and *Flaveria bidentis*, but exhibits distant relationships with rice, maize, and sweet sorghum. Notably, conserved motif analysis revealed interspecific divergence primarily within N-terminal regions across *Arabidopsis*, cassava, and maize *NADP-ME* families. *MeNADP-ME1* possessed a unique N-terminal motif (Motif 14), whereas *ZmchlC4NADP-ME* featured a distinctive N-terminal domain (Motif 5), and *ZmchlnonC4NADP-ME* lacked the N-terminal motif (Motif 15). These structural divergences correlate with the expansion of the *NADP-ME* gene family during the evolutionary trajectory toward C4 photosynthesis in cassava and maize. Furthermore, six segmentally duplicated gene pairs were identified among *MeNADP-ME* genes, with *MeNADP-ME3*/*MeNADP-ME4* (Ka/Ks > 1) originating under positive selection. Interspecies synteny analysis revealed that cassava shares fewer syntenic genes with monocots (rice, maize), whereas it exhibits greater synteny with eudicots (*Arabidopsis*, *Populus trichocarpa*). Notably, the highest number of syntenic genes was identified between cassava and its congeneric Euphorbiaceae species, the rubber tree. This finding further indicates the close evolutionary association of *NADP-ME* genes with species diversification and lineage divergence.

Cis-acting regulatory elements (CAREs) serve as specific functional modules on DNA sequences, which play critical roles in the dynamic regulation of gene expression [53,54]. Multiple CAREs associated with plant development and growth, hormone response, and stress response were identified in the upstream regions of cassava *NADP-ME* coding sequences. These types of elements are also commonly present within the upstream regions of *NADP-ME* genes in *Arabidopsis* and *Brassica napus* [33,55]. These findings indicate that *NADP-ME* genes exhibit broad responsiveness to external environmental stimuli and participate in mediating the regulation of gene expression and various physiological processes at the transcriptional level.

*MeNADP-ME* exhibits distinct expression patterns across different tissues and organs. Genes with high expression levels in specific tissues or organs are likely associated with particular physiological processes or functions crucial to cassava growth and development, potentially playing a vital role in these specific locations. The homologous genes *AtNADP-ME2* and *AtNADP-ME4* in *Arabidopsis* are involved in regulating plant organ development and growth and in responding to abiotic stress [23].

Single-cell RNA sequencing enables construction of high-resolution gene expression profiles at the cellular level, providing novel insights into key biological processes such as plant development and stress responses, and has emerged as a pivotal technique in modern plant research [56]. This study revealed that *MeNADP-ME3* exhibits relatively high expression levels in the bundle sheath cells of cassava leaves, suggesting the potential existence of a limited C4 photosynthetic pathway in cassava leaves. This observation aligns with the tissue-specific high expression pattern of *ZmchlC4NADP-ME* in bundle sheath cells observed in the typical C4 plant maize [57]. In contrast, within the C3 model plant *Arabidopsis*, expression of *AtcytNADP-ME1* is primarily localized to vascular cells, while *AtcytNADP-ME3* is expressed at higher levels within mesophyll cells [42]. Cross-species comparisons demonstrate that the expression pattern of *NADP-ME* genes in bundle sheath cells indicates that *MeNADP-ME3* likely fulfills a functional role analogous to its counterpart within the C4 photosynthetic pathway in cassava. Further single-cell clustering analysis revealed evolutionarily conserved expression patterns and functional adaptation of the *MeNADP-ME* gene family across distinct cell subsets. For instance, *MeNADP-ME1* and *MeNADP-ME4* are co-expressed at high levels in subsets of cells associated with substance transport in both cassava leaves and tuberous roots, suggesting potential synergistic involvement in metabolite translocation, whereas *MeNADP-ME2* is only minimally enriched in epidermal cells. These tissue-specific expression patterns not only reflect functional divergence and coordination among members of the *NADP-ME* gene family but also provide transcriptional regulatory evidence supporting their classification within a gene family possessing a shared evolutionary origin.

Cassava is characterized as a C3-C4 intermediate species that, although lacking complete Kranz anatomy, displays characteristic C4 traits such as high photosynthetic efficiency and low photorespiration [58]. Significantly, the activity of phosphoenolpyruvate carboxylase (PEPC), a key enzyme in the C4 pathway, is markedly higher in cassava than in typical C3 plants [5]. Plasmodesmata (PD), which serve as nanoscale conduits for intercellular transport in plants, play a pivotal role in the ${\mathrm{CO}}_{2}$ concentration mechanism (CCM) of C4 plants [59]. Previous studies identified mesophyll cells possessing transport functions surrounding bundle sheath (BS) cells in cassava [40]. Our scRNA-seq analysis of mesophyll cell subset 13 adjacent to BS cells and vascular tissue cell subsets revealed a co-upregulation of photosynthesis-related genes and PD-associated genes in both mesophyll subset 13 and BS cells. Furthermore, subcellular localization analysis indicated that MeNADP-ME3 localizes to the chloroplasts. These findings collectively suggest the presence of a limited C4 photosynthetic pathway in cassava. Protein–protein interaction prediction for MeNADP-ME3 identified malate synthase (MLS), an enzyme involved in photorespiration, as its highest-scoring interactor. This interaction provides a plausible molecular basis underlying cassava’s characteristic low photorespiration phenotype through potential regulatory mechanisms. Consequently, cassava serves as an ideal natural model system for investigating the evolutionary mechanisms of the C4 photosynthetic pathway, thereby holding significant value for deciphering plant adaptive evolution and the genetic improvement in stress resistance in crops.

C4 plants generally exhibit greater thermotolerance and lower photorespiration rates compared to C3 plants. This advantage primarily arises from their CCM, which sustains photosynthetic activity under low atmospheric ${\mathrm{CO}}_{2}$ concentrations induced by high-temperature-triggered stomatal closure. This mechanism suppresses the oxygenase activity of Rubisco, thereby minimizing energy-wasting photorespiration. Investigation of the MeNADP-ME gene family in cassava revealed distinct expression patterns under abiotic stresses. Most members were highly responsive to thermal extremes: transcript levels were significantly downregulated following cold stress yet universally upregulated under heat stress. This differential responsiveness aligns with cassava’s intrinsic attributes of heat tolerance, high photosynthetic efficiency, and low photorespiration. During PEG-simulated drought stress, *MeNADP-ME1* and *MeNADP-ME3* exhibited significant upregulation—a pattern consistent with NADP-ME responses in tobacco and wheat (*Triticum aestivum*) leaves [17,60]. Similarly, salt stress induced marked upregulation of *MeNADP-ME2* and *MeNADP-ME3*, corroborating cis-element predictions and echoing osmotic stress-responsive expression patterns observed in sweet sorghum [23]. The differential expression patterns of plant *NADP-ME* genes under environmental constraints reflect both their adaptive evolution during phylogeny and their functional specialization in mediating lineage-specific stress adaptation mechanisms.

Previous studies have validated the critical functional role of plastidic NADP-ME in plant stress responses [23,61]. In this study, we generated *Arabidopsis* transgenic lines overexpressing *MeNADP-ME3* with chloroplast-directed localization. Root elongation assays under diverse stress treatments demonstrated that primary root lengths of transgenic lines were significantly greater than WT counterparts under drought and salt stress, indicating the gene’s potential to sustain root physiological activity for growth maintenance under adversity. When exposed to environmental stressors, ROS accumulation induces oxidative damage, subsequently compromising cellular integrity through destruction of membrane systems, DNA, proteins, and lipids [62]. Malondialdehyde (MDA) is the final product of membrane peroxidation and serves as an index to measure the oxidative damage of plant cell membranes [63]. $H_{2}O_{2}$ (a predominant ROS in plants) acts both as a signaling molecule regulating physiological processes via aquaporin-mediated transmembrane transport and as an indicator of oxidative stress [64,65]. Transgenic lines exhibited significantly lower MDA content and $H_{2}O_{2}$ accumulation relative to WT following drought and salt treatments, demonstrating the pivotal function of *MeNADP-ME3* overexpression in mitigating oxidative injury.

Concurrently, activities of antioxidant enzymes (SOD, POD, CAT) were quantified. Superoxide dismutase (SOD) constitutes the primary defense by scavenging superoxide anions, while peroxidase (POD) and catalase (CAT) principally eliminate $H_{2}O_{2}$ [66]. Transgenic lines displayed significantly enhanced SOD, POD, and CAT activities compared to WT under both stress conditions. *MeNADP-ME3* may confer drought and salt tolerance in transgenic *Arabidopsis* through multiple mechanisms. It may indirectly upregulate antioxidant enzymes, thereby enhancing ROS scavenging and alleviating oxidative stress. Furthermore, it may promote osmotic adjustment and reduce membrane lipid peroxidation, as reflected by decreased MDA content. These combined effects ultimately improve the stress tolerance of transgenic lines.

## 5. Conclusions

This study reveals that the NADP-ME gene family in cassava is comparatively compact, with segmental duplication representing the primary evolutionary driving force. MeNADP-ME genes exhibit strikingly divergent expression profiles across different tissues and organs. Furthermore, their expression patterns within distinct cellular subpopulations in the single-cell RNA sequencing (scRNA-seq) demonstrate significant evolutionary conservation and functional adaptability. Subcellular localization confirmed MeNADP-ME3 targeting to chloroplasts in cassava leaves. ScRNA-seq analysis revealed its predominant expression in bundle sheath cells (BS), with an associated mesophyll subcluster 13 showing elevated transcription of photosynthesis-associated genes and plasmodesmata (PD)-related genes. These findings suggest the existence of a rudimentary limited C4 photosynthetic apparatus in cassava leaves. MeNADP-ME promoters harbor diverse cis-regulatory elements, indicating crucial roles in environmental adaptation, stress response, and developmental coordination. Differential expression analysis under environmental stresses identified *MeNADP-ME3* as markedly responsive to multiple abiotic stressors, suggesting its functional significance in stress adaptation. Overexpression of *MeNADP-ME3* in *Arabidopsis* was consequently performed for functional investigation. Under drought and salt stress, *MeNADP-ME3* overexpression likely potentiates antioxidant enzyme activity (SOD, POD, CAT) through indirect transcriptional regulation, thereby enhancing ROS scavenging efficacy. Concurrently, it augments osmoregulatory capacity and reduces membrane peroxidation, ultimately conferring elevated drought and salinity tolerance in transgenic lines. 

## Data Availability

The data presented in this study are available at https://www.ncbi.nlm.nih.gov/.

## Supplementary Materials

The following supporting information can be downloaded at: https://www.mdpi.com/article/10.3390/cimb48030331/s1.
